# 1231. Development of state public health goals for antimicrobial stewardship using local data

**DOI:** 10.1093/ofid/ofad500.1071

**Published:** 2023-11-27

**Authors:** Lauren Biehle, Rachel Schaefer, Christopher A Czaja

**Affiliations:** Colorado Department of Public Health and Environment, Denver, Colorado; Colorado Department of Public Health and Environment, Denver, Colorado; Colorado Department of Public Health and Environment, Denver, Colorado

## Abstract

**Background:**

Antimicrobial stewardship (AS) is critical to effectively treat infections and combat antibiotic resistance. Our Healthcare-Associated Infections/Antimicrobial Resistance (HAI/AR) program used state-level data to set public health priorities for AS across diverse acute care (ACH) and critical access (CAH) hospital settings.

**Figure:**
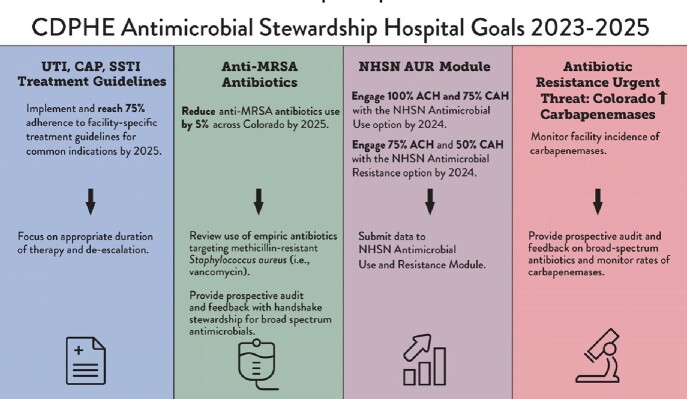

Infographic describing antimicrobial stewardship goals and priority actions for Colorado hospitals

**Methods:**

Hospitals reported antibiotic use (AU) data and implementation of priority core elements of AS to the National Healthcare Safety Network (NHSN). The Colorado Department of Public Health and Environment (CDPHE) accessed the data according to a data use agreement with NHSN. We used these data and antibiotic resistance surveillance data to inform state public health goals for AS.

**Results:**

AU data were available for 47/53 ACH (88%) and 18/31 (58%) CAH. Three antibiotics (ABS) of highest use were ceftriaxone (18% of all ABS reported), vancomycin (14%), and piperacillin-tazobactam (10%). Data from the NHSN Patient Safety Survey indicated prospective audit and feedback (PAF) occurred in 79% of ACH and 47% of CAH. Monitoring discharge duration of therapy was reported in 27% of ACH and 37% of CAH. We developed three goals: Goal One is to decrease broad-spectrum ABS used for community-acquired infections (BSCA) by 5% in five years by encouraging hospitals to achieve 75% adherence to facility-specific treatment guidelines by 2025. Goal Two is to reduce the use of ABS active against methicillin-resistant *Staphylococcus aureus* (MRSA) by 5% in five years by encouraging facilities to evaluate their empiric anti-MRSA ABS and provide PAF. Goal Three is to increase NHSN AU/AR reporting to 100%/75% of ACH and 75%/50% of CAH with support through virtual AU/AR office hours and financial grants. Finally, we identified an increase in carbapenemases as an urgent threat and will encourage facilities to review use of broad-spectrum ABS and provide PAF, while monitoring rates of carbapenemases. These initiatives will be promoted through AS collaborations and feedback reports, with progress monitored through the AU Targeted Assessment for Stewardship reports.

**Conclusion:**

These novel state-wide AS initiatives are tailored by local data and best practices to support a common vision for action for hospital AS providers.

**Disclosures:**

**All Authors**: No reported disclosures

